# Oxidative Stress Biomarkers as a Predictor of Stage Illness and Clinical Course of Schizophrenia

**DOI:** 10.3389/fpsyt.2021.728986

**Published:** 2021-11-15

**Authors:** Dariusz Juchnowicz, Michał Dzikowski, Joanna Rog, Napoleon Waszkiewicz, Anna Zalewska, Mateusz Maciejczyk, Hanna Karakuła-Juchnowicz

**Affiliations:** ^1^Department of Psychiatric Nursing, Medical University of Lublin, Lublin, Poland; ^2^Department of Psychiatry, Psychotherapy and Early Intervention, Medical University of Lublin, Lublin, Poland; ^3^Department of Psychiatry, Medical University of Bialystok, Choroszcz, Poland; ^4^Experimental Dentistry Laboratory and Department of Restorative Dentistry, Medical University of Bialystok, Bialystok, Poland; ^5^Department of Hygiene, Epidemiology and Ergonomics, Medical University of Bialystok, Bialystok, Poland

**Keywords:** schizophrenia, oxidative stress, antioxidant, psychosis, biomarkers

## Abstract

Pro/antioxidant imbalance has been reported in schizophrenia (SZ). However, the results of studies are inconsistent and usually do not include other factors that are highly affected by oxidative stress (OS).This cross-sectional study aimed to determine the serum levels of OS markers and their potential connection with schizophrenia. The total sample comprised 147: 98 individuals with SZ −47 first-episode (FS) and 49 chronic patients (CS)—and 49 healthy individuals (HC) as a control group. The examination included clinical variables and serum levels of antioxidants and oxidative damage products. The significant changes were observed in concentrations of all examined markers, without any specific direction of the pro/antioxidant balance shift between SZ and HC. In the regression model adjusted for cofounders, catalase: OR = 0.81 (95%CI: 0.74–0.88); glutathione peroxidase: OR = 1.06 (95%CI: 1.02–1.10); total antioxidant capacity: OR = 0.85 (95%CI: 0.75–0.98); oxidative stress index: OR = 1.25 (95%CI: 1.03–1.52); ferric reducing ability of plasma: OR = 0.79 (95%CI: 0.69–0.89); advanced glycation end products: OR = 1.03 (95%CI: 1.01–1.04); and advanced oxidation protein products (AOPP): OR = 1.05 (95%CI: 1.03–1.07) turned out to be significant predictors of schizophrenia. In the multiple stepwise regression model, pro/antioxidant status and their interaction with the duration of illness-related factors affected schizophrenia symptoms: positive symptoms (FRAPxKYN), negative (DITYR, FRAP, CAT), general (KYN), and over-all psychopathology (KYNxNFK). The results confirm differences in serum levels of oxidative biomarkers between SZ patients and healthy individuals. The pro/antioxidant status could be considered a predictor of schizophrenia and the factor affects patients' symptom severity.

## Introduction

Schizophrenia (SZ) affected ~ 1% around the world and, according to the World Health Organization (WHO), was ranked as one of the top 10 illnesses contributing to the global burden of disease (1). Despite the growing knowledge of the disease, the mechanisms underlying them are still poorly understood, but more and more evidence confirms no one contributory cause. To an increasing extent, SZ has been seen as a manifestation of the interplay between many pathophysiological processes affecting not only the brain but also other organs (2, 3).

One of the factors involved in the pathophysiology of SZ is oxidative stress (OS). OS is an imbalance between oxidative and reductive reactions, increasing reactive oxygen species (ROS), and decreasing antioxidant levels. Brain cells are highly vulnerable to OS due to a high metabolic rate (use of oxygen) and modest levels of antioxidants following a high abundance of peroxide substrates (4). ROS can come from the inactivation of monoaminergic neurotransmitters, as by-products of adrenaline, noradrenaline, dopamine, and serotonin (5). On the other hand, ROS are the secondary messengers, fundamental in many brain-connected processes, including cell growth, differentiation, and proliferation; signaling; and immune response. There is also some evidence, stating that ROS are engaged in human adult neurogenesis (5). Therefore, ROS has both pro-survival and pro-death effects, and their excess leads to OS and inflammation promotion. The imbalance between antioxidant and pro-oxidant could negatively affect neural growth, differentiation, regeneration, and synaptic plasticity. OS may play a role in the neuroprogression of SZ *via* DNA damage and immune-inflammatory pathways (6). Experimental evidence confirms that OS is involved in apoptotic pathways mediating the inflammatory response of CNS. Indicators of OS could be released by activation of immune cells and vice versa—OS can activate an immune-inflammatory pathway (7).

Consequently, SZ should be considered in the context of CNS dysfunction and other organ changes, including the interplay between the imbalance of pro/antioxidant state. Some of the oxidative-related factors have been proposed as potential biomarkers of SZ (8, 9). Although there are failures to replicate results, both observational and intervention studies support the association between redox imbalance and SZ. Most studies assessing OS in SZ populations point to an increase of toxic by-products due to both increased pro-oxidants and decreased antioxidants (10–12). Several hypotheses concerning the oxidative pathophysiology of SZ have been postulated. However, the source of abnormalities remains unclear (13). Despite the extensive studies focusing on indicators of OS, there is no clear consensus of the interplay between oxidation markers and SZ.

Considering the aforementioned, our study aimed to quantify the serum levels of OS markers and their potential connection with SZ.

## Materials and Methods

### Study Population

The study included 147 participants aged 18–65 years: 98 patients that met the criteria of SZ according to the Diagnostic and Statistical Manual of Mental Disorders, Fifth Edition (DSM-5) (14) and 49 healthy individuals (HC) as a control group. Among the SZ group, 49 were the first-episode patients (FS, up to 24 months after first treatment contact), and 49 were chronic patients (CS).

Exclusion criteria were as follows: co-occurrence of neurological diseases, intellectual disability, organic brain dysfunction, autoimmune diseases or other diseases in unstable phase (including metabolic diseases) or addiction (except nicotine and caffeine), the present clinical signs of inflammation (hsCRP ≧ 5 μg/ml) and/or leukocytosis (>10.000 G/I) during entry to the study, and having psychiatric diagnosis (in the past or current) in HC group.

All participants gave written informed consent. The study was conducted in accordance with the Declaration of Helsinki, and the protocol was approved by the Ethics Committee of the Medical University of Lublin, Poland (project identification code: KE-0254/231/2013).

### Blood Collection

Venous blood (20 ml) samples were collected after overnight (12-h) fasting and centrifugation (at 2,000 × g, 10 min at room temperature). The obtained serum has been stored at −80°C and thawed only once to further analysis.

### Laboratory Tests

#### OS Biomarkers

The pro/antioxidant balance assessment included the following: 1) enzymatic and non-enzymatic antioxidants: catalase (CAT), glutathione peroxidase (GPx), superoxide dismutase-1 (SOD-1), glutathione reductase (GR), reduced glutathione (GSH), total antioxidant capacity (TAC), and ferric reducing ability of plasma (FRAP) and 2) oxidative damage products: advanced glycation end products (AGEs), advanced oxidation protein products (AOPP), dityrosine (DITYR), kynurenine (KYN), N-formylkynurenine (NFK), tryptophan (TRY), and total oxidant status (TOS), as well as concentrations of nitric oxide (NO) and total protein. All reagents for the biochemical assays (unless otherwise specified) were obtained from Sigma-Aldrich, Germany. The absorbance/fluorescence was measured using Infinite M200 PRO Multimode Microplate Reader (Tecan, Mannedorf, Switzerland). All determinations were estimated in duplicate samples and standardized to 100 mg of total protein.

#### Enzymatic and Non-enzymatic Antioxidants

Seven indicators of antioxidant defense were assessed:

1. The activity of CAT (EC 1.11.1.6) was determined colorimetrically by measuring the decomposition rate of hydrogen peroxide in a 50-mM phosphate buffer (pH 7.0) at 240 nm (15). One unit of CAT activity was defined as the amount of enzyme that decomposes 1 mmol hydrogen peroxide for 1 min.
2. The activity of SOD-1 (E.C. 1.15.1.1) was assayed colorimetrically by measuring the cytosolic activity of SOD by inhibiting the oxidation of adrenaline to adrenochrome in pH 10.2 at 480 nm (16). It was assumed that one unit of SOD activity inhibits the oxidation of adrenaline by 50%.
3. The activity of GPx (EC 1.11.1.9) was measured colorimetrically based on the reduction of organic peroxides by GPx in the presence of NADPH and glutathione reductase (GR) at 340 nm (17). One unit of GPx activity was assumed to catalyze oxidation of 1 μmol of NADPH for 1 min.
4. The activity of GR (EC 1.6.4.2.) was determined colorimetrically by measuring the decrease in absorbance of NADPH at 340 nm (18). One unit of GR activity was defined as that quantity of enzyme which catalyzes the oxidation of 1 μmol of NADPH for 1 min.
5. The concentration of GSH was analyzed colorimetrically by reaction with 5,5′-dithiobis-2-nitrobenzoic acid (DTNB) in a 0.2-M phosphate buffer (pH 8.0) to give a complex that absorbs at 412 nm (19).
6. TAC was estimated colorimetrically using two, 2-azinobis-3-ethylbenzothiazoline-6-sulfonic acid radical cation (ABTS^*+^) (20). Changes in the absorbance were determined at 660 nm, and the TAC level was calculated from the calibration curve for 6-hydroxy-2,5,7,8-tetramethylchroman-2-carboxylic acid (Trolox).
7. FRAP was determined colorimetrically by measuring the ferric reducing ability of samples with TPTZ (2, 4, 6-tripyridyl-s-triazine) (21). Changes in the absorbance were determined at 593 nm, and FRAP level was calculated from the calibration curve for iron sulfate (FeSO_4_).

#### Oxidative Damage Products

We chose six biomarkers related to oxidative damage in SZ for examination:

1. The content of AGEs was analyzed fluorimetrically by measuring AGE-specific fluorescence emission/excitation at 350 nm/440 nm (22). For AGE determination, serum samples were diluted 1:50 (*v:v*) in phosphate-buffered saline (PBS, pH 7.2).
2. The concentration of AOPP was assayed colorimetrically by measuring the oxidative capacity of the iodine ion at 340 nm (22). For AOPP determination, serum samples were diluted in PBS (pH 7.2) 1:50 (*v:v*).
3. To detect DITYR, KYN, NFK, and TRY, samples were diluted 1:10 (*v:v*), in 0.1 M H_2_SO_4_, and fluorescence at 330/415, 365/480, 325/434, and 95/340 nm, respectively, was measured (23). The results were normalized to fluorescence of 0.1 mg/ml quinine sulfate in 0.1 M H_2_SO_4_ (24).
4. The concentration of TOS was estimated colorimetrically based on the oxidation of Fe^2+^ to Fe^3+^ ions in the presence of the oxidants contained in the sample (25). The absorbance was measured bichromatically at 560/800 nm. The oxidative stress index (OSI) was calculated using the formula: OSI = TOS/TAC *x* 100 (23).
5. The level of NO was determined colorimetrically by measuring its stable decomposition products ${\text{NO}}_{3}^{-}$ and ${\text{NO}}_{2}^{-}$ by the Griess reaction (26). Changes in the optical density were analyzed at 543 nm.
6. The concentration of total protein was measured by the bicinchoninic acid (BCA) method (27) using commercial kit Thermo Scientific Pierce BCA Protein Assay (Rockford, IL, USA) following the manufacturer's instructions.

### Sociodemographic and Clinical Data

The characteristics of the study sample were determined using a self-constructed questionnaire including sociodemographic/lifestyle information, including duration of illness (DoI), number of episodes (NoE), medication, comorbidity, number of hospitalizations (NoH), and number of smoking cigarettes daily. The clinical data on patients were obtained from a supervising physician during the blood collection day. The doses of antipsychotic medication were calculated based on defined daily doses (DDDs) to 1 mg olanzapine (28). The severity of SZ symptoms was assessed by a well-trained physician (M.D.) using the Polish adaptation of the Positive and Negative Symptom Scale (PANSS) (29).

### Statistical Analysis

We used Statistica 13 software (TIBCO Software Inc., Palo Alto, CA, USA) to analyze obtained results. To determine the distribution of variables, the Shapiro–Wilk test was applied. Due to non-Gaussian distribution in most of the factors, non-parametric tests were used: Man–Whitney U to determine the differences between two groups and Kruskal–Wallis test in three groups for continuous variables. To establish the differences between categorical variables across the group, a chi-square test was applied. We used Spearman's rho correlation to determine the magnitude and direction of the correlation. For all analyses, *p* < 0.05 was considered statistically significant. We used Bonferroni correction for *post hoc* correction to control for type I error.

We performed univariable logistic regression to assess the potential odds of SZ depending on OS. The odds ratio and 95% confidence interval for markers which was statistically significant were determined. We also adjusted the obtained models of potential cofounders in the multiple analysis with interactions.

To assess the possible effect of oxidative status on psychopathological symptoms, multiple stepwise regression analysis with automatic selection of variables was conducted. The obtained models were verified by determination of the adjusted *R*-squared. We included the following variables as potential predictors: age, number of hospitalization, duration of illness, olanzapine equivalents (OE), gender, body mass index (BMI), and number of smoking cigarettes were included in the model as possible confounders.

## Results

### Characteristics of the Study Population

The characteristics of the examined population are shown in Table 1. The study group included 147 participants: 98 individuals with SZ (SZ group) divided into two subgroups—chronic (CS; *n* = 49) and first-episode patients (FS; *n* = 47)—and 49 healthy individuals constituting the control group (HC). There was a difference in the age of chronic and first-episode patients (*p* < 0.001). The CS group had a higher BMI than the SZ group (*p* = 0.03) and HC (*p* < 0.001). Not surprisingly, chronic patients had a longer length of illness, more psychotic episodes (*p* < 0.001), and lower severity of SZ symptoms (*p* < 0.001) compared to the FS group. The median of smoking cigarettes per day was 0 across all examined groups (*p* > 0.05). Fifteen participants from the CS group (30.61%) and two participants from the FS group (4.08%) received typical antipsychotic drugs. Fifteen FS (30.61%) and 43 CS (87.76%) patients received atypical antipsychotics drugs. Other individuals from the FS group were drug-naive.

**Table 1:** Characteristic of examined population.

**Variable**	**SZ**	**FS**	**CS**	**HC**	***p*-value**
Age, years [M]	29	21^1^	40^1^	28^1^	^1^ <0.001
Gender (male) [%]	52 (53.06)	26 (53.06)	26 (53.06)	21 (42.86)	NS
BMI, kg/m^2^ [M]	24.5	21.3^2^	25.9^1/2^	22.9^1^	^1^0.03 ^2^ <0.001
Smoking cigarettes per day [M]	0	0	0	0	NS
Duration of illness, months [M]	24	3^1^	241^1^	NA	^1^ <0.001
Number of episodes [M]	3	1^1^	7^1^	NA	^1^ <0.001
PANSS total [M]	87	104^1^	72^1^	NA	^1^ <0.001

*SZ, schizophrenia; FS, first episode; CS, chronic schizophrenia; HC, healthy control; BMI, body mass index; PANSS, Positive and Negative Symptoms Scale*.

1, 2 *indicate groups with differences. M, median*.

### The Differences in OS Biomarkers Between Patients With SZ and Healthy Individuals

The obtained results confirm substantial differences in redox state between individuals who have SZ and healthy persons (see Table 2). The significant changes were observed in concentrations of CAT [higher in HC and lower in FS group (*p* < 0.001)], SOD which was the lowest in chronic patients and the highest in healthy control (*p* < 0.006), and GSH (which was substantially higher in healthy persons compared to the all-patient group *p* < 0.001). Compared to healthy individuals, the changes in GPx concentrations were confirmed in the CS (*p* = 0.012) and SZ (*p* = 0.018) groups but not in the FS group. The GR reduction was noted in FS patients compared to CS (*p* = 0.003). The TAC (SZ>HC; FS < CS; CS>HC) was higher in the CS group (*p* < 0.001), and TOS, OSI, FRAP, and AGEs differed in healthy individuals compared to the all-examined-patients group (lower levels of TOS, OSI, AGEs, and AOPP) and higher levels of FRAP in the HC group (*p* < 0.001). AOPP also had a different concentration in serum of first-episode and chronic patients (FS < CS; *p* = 0.025). The greater levels characterized FS and HC groups compared to CS (*p* = 0.001 and *p* = 0.002, respectively). A higher concentration of KYN was noted in the HC group compared to SZ (*p* = 0.002) and the FS group (*p* < 0.001). KYN was also higher in first-episode patients compared to the chronic group (*p* < 0.001). NFK differentiated SZ (*p* = 0.003) and CS patients (*p* < 0.001) compared to HC (lower in patients groups) but was also lower in CS compared to FS patients (*p* = 0.001). The lower concentration of NO was noted in the FS group compared to CS (*p* = 0.013) and HC groups (*p* = 0.046).

**Table 2:** The differences in oxidative stress between examined groups.

**Variables**	**SZ**	**FS**	**CS**	**HC**	***p*-value**
**Antioxidant defense biomarkers**
CAT	26.77^1^	30.14^2^	25.23^3^	41.21^1/2/3^	^1/2/3^ <0.001
GPx	36.94^1^	36.21	37.632^2^	33.07^1/2^	^1^0.018 ^2^0.012
SOD	203.75	289.38^1/2^	145.16^1/3^	217.39^2/3^	^1^ <0.001 ^2^0.036 ^3^ <0.006
GR	1.11	1.04^1^	1.33^1^	1.08	^1^0.003
GSH	0.17^1^	0.17^2^	0.17^3^	0.38^1/2/3^	^1/2/3^ <0.001
TAC	53.67^1^	47.90^2^	60.20^2/3^	46.82^1/3^	^1/2/3^ <0.001
TOS	1777.48^1^	1680.57^2^	1826.37^3^	189.41^1/2/3^	^1/2/3^ <0.001
OSI	32.02^1^	33.07^2^	30.93^3^	4.44^1/2/3^	^1/2/3^ <0.001
FRAP	11.96^1^	13.90^2^	11.27^3^	15.79^1/2/3^	^1/3^ <0.001 ^2^0.004
**Oxidative damage products**
AGEs	420.84^1^	414.98^2^	424.32^3^	379.15^1/2/3^	^1/2/3^ <0.001
AOPP	1.15^1^	1.01^2/3^	1.23^3/4^	0.53^1/2/4^	^1/2/4^ <0.001 ^3^0.025
DITYR	851.68	895.47^1^	822.86^1/2^	892.86^2^	^1^0.001 ^2^0.002
KYN	715.63^1^	785.94^2^	452.53^2/3^	793.64^1/3^	^1^0.002 ^2/3^ <0.001
NFK	307.45^1^	330.65^2^	226.90^2/3^	343.35^1/3^	^1^0.003 ^2^0.001 ^3^ <0.001
TRY	2754.19	2765.01	2713.05	2785.31	NS
NO	1.79	1.49^1/2^	2.40^1^	2.12^2^	^1^0.013 ^2^0.046

*SZ, schizophrenia; FS, first episode; CS, chronic schizophrenia; HC, healthy control; CAT, catalase; GPx, glutathione peroxidase; SOD-1, superoxide dismutase-1; GR, glutathione reductase; GSH, reduced glutathione; TAC, total antioxidant capacity; TOS, total oxidant status; OSI, oxidative stress index; FRAP, ferric reducing ability of plasma; AGEs, advanced glycation end products; AOPP, advanced oxidation protein products; DITYR, dityrosine; KYN, kynurenine; NFK, N-formylkynurenine; TRY, tryptophan; NO, nitric oxide*.

1, 2, 3, 4 *indicate groups with differences. Data are presented as median*.

Some of the indicated results turned out to be not significant after Bonferroni correction (GPx between all patients with SZ and HC, SOD-1 between FS and HC, AOPP between FS and CS, NO between FS and HC).

In the analysis of gender effect on OS status, we found higher TOS in CS males compared to females (*p* < 0.05) and higher SOD in HC males compared to females (*p* < 0.05).

### The Relationship Between OS and Lifestyle/Clinical Features Across Study Groups

We found many correlations between OS and health- and illness-related parameters. However, most of them were weak (see Supplementary Table 1). A strong association was only seen between BMI and SOD-1 concentration in the HC group (*R* = −0.55; *p* < 0.05). It is worth mentioning that the relationships between SOD-1, age (*R* = −0.49; *p* < 0.05), DoI (*R* = −0.47; *p* < 0.05); TAC, DoI (*R* = 0.47; *p* < 0.05); and KYN, age (*R* = −0.50; *p* < 0.05), DoI (*R* = −0.48; *p* < 0.05) in the SZ group were achieved between moderate to large difference effects.

### The pro/Antioxidant Imbalance and Odds of SZ

#### Univariate Analysis

The results of the univariate analysis are shown in Table 3. Higher concentrations of GPx, TAC, OSI, AGEs, and AOPP had increased odds of SZ. Lower concentrations of CAT, GSG, and FRAP were also positively connected with a SZ diagnosis.

**Table 3:** Odds ratio between schizophrenia and oxidative stress: crude results.

	**OR**	**95% CI**	***p*-value**
CAT	0.84	0.79–0.90	<0.0001
GPx	1.06	1.02–1.10	0.002
GSH	0.84	0.78–0.89	<0.0001
TAC	1.06	1.03–1.10	0.0004
OSI	1.11	1.06–1.15	<0.0001
FRAP	0.76	0.67–0.86	<0.0001
AGEs	1.03	1.01–1.04	<0.0001
AOPP	1.05	1.03–1.06	<0.0001

*CAT, catalase; GPx, glutathione peroxidase; GSH, reduced glutathione; TAC, total antioxidant capacity; OSI, oxidative stress index; FRAP, ferric reducing ability of plasma; AGEs, advanced glycation end products; AOPP, advanced oxidation protein products; OR, odds ratio; CI, confidence interval*.

#### Multivariable Analysis

In the model adjusted for BMI, number of cigarettes and age CAT: OR = 0.81 (95%CI: 0.74–0.88); GPx: OR = 1.06 (95%CI: 1.02–1.10); TAC: OR = 0.85 (95%CI: 0.75–0.98); OSI: OR = 1.25 (95%CI: 1.03–1.52); FRAP: OR = 0.79 (95%CI: 0.69–0.89), AGEs: OR = 1.03 (95%CI: 1.01–1.04); and AOPP: OR = 1.05 (95%CI: 1.03–1.07) remained significant predictors of SZ.

### The Effect of Pro/Antioxidant Imbalance on SZ Symptoms

In the forward multiple stepwise regression model, the positive symptoms were lowered with the DoI and were increased with simultaneous FRAP and KYN rising (see Table 4). The following model was adjusted for FRAP and KYN concentration and explained the 24.55% variability of positive symptoms in the SZ group.

**Table 4:** The oxidation-related predictors of psychopathological symptoms in schizophrenia: multiple models.

**Positive symptoms**	***R* ^**2**^**	***p*-value**	**β**
FRAPxKYN	24.55%	0.03	0.93
DoI		0.002	−0.36
**Negative symptoms**	***R*** ^**2**^	***p*** **-value**	**B**
NoH	28.73%	0.006	2.33
DITYR		<0.001	0.50
NoHxDITYR		0.01	−2.09
FRAP		0.04	−0.20
CAT		0.004	−0.28
**General symptoms**	***R*** ^**2**^	***p*** **-value**	**B**
NoH	45.78%	0.03	0.28
DoIxKYN		0.006	−0.63
**Psychopathological symptoms (total PANSS)**	***R*** ^**2**^	***p*** **-value**	**B**
KYNxNFK	34.42%	0.03	1.22

*CAT, catalase; FRAP, ferric reducing ability of plasma; KYN, kynurenine; NFK, N-formylkynurenine; DoI, duration of illness; NoH, number of hospitalization; R^2^, coefficient of determination; ?, standardized regression coefficients beta; x, interaction between variables*.

With NoH, DITYR concentration, and interaction between FRAP and CAT, their concentrations turned out to be factors that affect negative symptoms in the backward multiple stepwise regression model. The following model explained 28.73% variability of negative symptoms in the SZ group.

The general symptoms were positively affected by the NoH and negatively affected by the higher concentration of KYN and longer DoI as one in the model adjusted for OE, NFK, and interaction between them, and KYN and interaction between KYN and NFK, DoI. This forward multiple-step regression model explained the 45.78% variability of general symptoms in the SZ group.

In the multiple stepwise forward regression, simultaneous increases in KYN and NFK concentrations affected psychopathological symptoms (total PANSS score) in the model adjusted for DoI and interaction between it and KYN, KYN, and NFK. The following model explained the 34.42% variability of symptoms in the SZ group.

In the FES group, the higher severity negative symptoms were related to lower CAT concentration in the model adjusted for DITYR, NFK, and interaction between them (multiple stepwise forward regression model). The following model explained 24.56% of the variability of negative symptoms in the FS group.

## Discussion

The role of OS in psychiatric disorders and SZ remains unclear. The meta-analysis published in 2019 confirmed that some of the markers were different in the patients' group than healthy individuals. Nevertheless, CAT (blood cells), GPx (blood cells, serum/plasma), GSH (serum/plasma), SOD (blood cells), SOD (serum/plasma) concentrations were unaffected, while TAS was the only marker that was lower in FES (11). Nevertheless, the authors did not adjust the results for many important disease-related factors, including the type or dose of antipsychotics. It should be pointed out that the definition of FEP varied across studied populations, patients on a different stage of illness may be assigned to this group, and diagnostic classification is not well-designed to facilitate biological and biochemical differentiation (11).

We found many indicators of redox imbalance in SZ patients. Some of the antioxidants were raised, while others decreased compared to healthy individuals. These ambiguous results may be the effect of other mechanisms that strongly affect OS and antioxidant defense of cells or psychotropic drugs and biological adaptation to illness.

In our study, the CAT concentration was negatively linked to the duration of illness (*R* = −0.29, *p* < 0.05) and the number of episodes (*R* = −0.31, *p* < 0.05). What is more, higher levels were related to a lower chance of SZ and negative symptoms. Taken together, as the disease progresses, the antioxidant enzyme stores are likely to run out. In two meta-analyses, CAT was not different between healthy individuals and the first-episode SZ patients (11, 12). CAT converts H_2_O_2_ into H_2_O and O_2_. There was also the hypothesis that polymorphism of the CAT gene could be engaged in earlier SZ development in males (30).

According to our results, the GPx concentration increased in chronic and first-episode patients compared to healthy individuals. Moreover, higher GPx was related with higher SZ chance (OR = 1.06; 95% CI: 1.02–1.10, *p* = 0.002). GPx levels may rise in the adaptive mechanism due to increased OS at the first episode of psychosis. Still, differences between HC and FS in GPx concentrations were not noted. In the meta-analysis, FEP was also no different from CAT from healthy individuals and patients with different SZ subtypes (11, 12). GPx is an antioxidant that, together with CAT, converts H_2_O_2_ into H_2_O and O_2_. Based on two meta-analysis results, differences in GPx concentration between SZ and healthy individuals were not seen (12). However, it was shown that in acutely relapsed and chronic inpatients subgroups GPx in RBC and serum were significantly decreased (10). The results in FEP individuals were discordant (10, 11).

The SOD-1 concentration was lower in chronic SZ but not in the first episode compared to healthy individuals. The potential SOD-1 role in SZ chance was not noted. In our study, the concentration of SOD-1 turned out to be related to psychopathological symptoms. These results suggest the role of SOD-1 in diminished OS by the interaction with peroxidation compounds. The meta-analysis indicated that SOD-1 activity significantly decreased in the disorganized type of SZ (12). The SOD-1 role is to catalyze the conversion of superoxide radicals to hydrogen peroxide. SOD-1 was to shown to be decreased in acutely relapsed inpatients (RBC), stable medicated out (RBC) patients, and FEP (RBC) (10). SOD-1 increased in stable medicated outpatients (serum) and chronic inpatients (RBC). In one meta-analysis in FEP, levels of SOD were increased in plasma but decreased in RBC, and in a Turkish study, the polymorphism of gene SOD was related to an increased risk of SZ. The observed reduced SOD activity should increase superoxide radicals (10), but a later meta-analysis did not support this (11).

In our study, the GR concentration was lower only in FS patients compared to the CS group. The GR fall with the time since diagnosis was confirmed by the correlation between GR and both the duration of illness (*R* = 0.35; *p* < 0.05) and the number of episodes (*R* = 0.31; *p* < 0.05) in SZ patients. However, we did not see the directly GR effect on symptoms or SZ chance.

GSH concentration was lower across all patients' groups, independent of illness stage. Their level was related to lower SZ chance, but this was not significant after adjusting for health-related factors. In the meta-analysis from 2019, there were no changes in GSH in FEP group serum and plasma (11).

It is well-known that assessment of redox homeostasis cannot be based solely on single biomarkers. Much more information about the efficiency of the antioxidant barrier is provided by the analysis of the total antioxidant potential. TAC takes into account the interactions between individual antioxidants (31, 32). TAC was different between patients and HC. The higher levels were in CS compared to both FEP and HC. The higher levels of TAC were related to SZ diagnosis chance. Surprisingly, after covariates were included, TAC was a factor inversely related to SZ odds. Some studies indicate no differences in TAC levels between patients and healthy individuals (33).

Although we did not directly assess the rate of ROS production, a shift in redox homeostasis in favor of oxidative reactions may be evidenced by elevated TOS. This parameter expresses the total content of oxidants in a biological system (25, 34). TOS was ~ 10-fold in SZ (independent on stage disease) compared to healthy individuals, and in the multivariable analysis, higher TOS increased SZ risk. Nonetheless, the concentration was not related to psychopathological symptoms. In opposition to our results, the concentration of TAS in serum and plasma was significantly decreased during the first episode, according to two meta-analyses (10, 11).

Also, OSI was several times higher in all SZ patients than HC, and rising levels increased SZ chance by 25% [OR = 1.25 (1.03–1.52)]. Most studies supported these results in chronic patients (35–37), and OSI was found to be higher in deficit SZ and remitted SZ patients compared to non-deficit and non-remission ones (38, 39).

We found a lower concentration of FRAP in both the first-episode and chronic patients, but a more significant difference was occurring between chronic patients and healthy individuals. Other studies supported it (40, 41); one study suggests that FRAP was lower in first-episode drug-naïve patients compared to healthy control (42). According to our study, lower FRAP concentration was a predictor of SZ risk after lifestyle variable adjustment [OR = 0.79 (0.69–0.89)], and simultaneous increases of FRAP and KYN were related to higher severity of positive symptoms in the multivariable analysis. In the multiple models, higher levels of FRAP affected the lower severity of negative symptoms, which is supported by authors who had observed a trend toward an inverse correlation between negative symptoms and FRAP (42). Lower FRAP was suggested to engage in cognitive deficits (36). This indicates the role of FRAP in the interaction with other oxidative compounds and the enzyme role in attempt reduction OS during psychosis development followed by depletion with the duration of illness. The longitudinal study suggests increasing in FRAP levels during antipsychotic treatment (41).

AGEs which turned out to be a predictor of SZ was higher in the patients' group, especially chronic patients, than healthy individuals. A consequence of disruption of antioxidant systems is intensified oxidation and glycation of proteins and lipids. Therefore, the increase in oxidative and carbonyl stress biomarkers in the progression of SZ is not surprising. The review conducted in 2015 showed that AGE accumulation is enhanced in subjects with SZ (43). The longitudinal study confirmed increased AGE accumulation in the skin, implicating a growing cardiovascular risk in psychosis (44).

AOPP, the marker of protein damage induced by oxidation stress, was elevated in all patients, with the most substantial rising in the chronic group. Our results are not supported by all studies (45), but some confirm that AOPP was increased in schizophrenic patients and suggest that AOPP could be considered an independent risk factor for SZ (46). Their potential rising with the duration of illness was suggested by relationship to age (*R* = −0.39; *p* < 0.05) and duration of illness (*R* = −0.35; p <0.05) in chronic patients. The AOPP increased the risk for SZ, and the connection remained significant after analyzing the covariates. There was also the suggestion that deficit SZ is characterized by higher AOPP levels (47). The assessment of drug-free patients has noticed that the levels of AOPP were related to higher severity of general symptoms and total psychopathology (46), but we did not find any effect of AOPP on SZ symptoms.

The higher DITYR (product of oxidative damage) was seen in first-episode patients and healthy individuals compared to the CS group. Formulated DITYR residues can increase AOPP production. The lower DITYR in chronic patients could be the result of failure to detect them due to excessive AOPP production. In the SZ group, there was also a correlation between psychopathological symptoms and DITYR levels: positive (*R* = 0.29; *p* < 0.05), general (*R* = 0.31; *p* < 0.05), and total psychopathology (*R* = 0.31; *p* < 0.05). It should be pointed out that multivariable analysis confirmed the relationship between DITYR and negative symptoms only.

Despite the noticed relationship between KYN and age in the SZ group, the HC and SZ group did not differ in age but had different KYN and NFK concentrations. We also found the correlation between positive (*R* = 0.43; *p* < 0.05), general (*R* = 0.39 *p* < 0.05), all symptoms (*R* = 0.38; *p* < 0.05), and KYN. Our multivariable regression analysis suggests the interaction between KYN and NFK in the induction of negative symptoms and the interplay between FRAP and KYN in the severity of positive symptoms. Kynurenine metabolites can cross the blood–brain barrier. However, the relevance of metabolites within peripheral circulation is not fully understood, but the substances are involved in the inflammatory response of CNS (48).

The higher NO concentration was seen only in chronic compared to first-episode patients. The obtained results could be affected by antipsychotic treatment, confirmed by a meta-analysis showing that only patients under treatment have higher levels of plasma/serum nitric oxide than controls (49). Nitric oxide possesses properties of both pro- and antioxidants. It can also stimulate the peroxidation of lipids and mediate antioxidant reactions in cellular membranes (49). In our study, NO concentration was related to age, except in the SZ group only, which suggests that this relationship is spurious (*R* = −0.35; *p* < 0.05).

Studies on OS in SZ are still at the beginning, especially regarding state-specific factors (specific to acute phases, first episode, or early stages), and the psychiatric population results are often contradictory. Inconsistent findings might be the result of different types of collected samples (serum, whole blood, mRNA, brain tissue), method of measurement, stages of illness, patients' treatment, comorbidity, and lifestyle factors (including diet and physical activity) (7, 12, 42). OS can lead to macromolecule oxidation, disruption of cell behavior, and conditions (50). Studies suggest that peripheral OS operates in a synchronized way with central response (51).

Further studies are needed to clearly define the role of OS in individuals with psychosis regarding state-specific factors (to acute phases, first episode, or early stages) and which of them are more durable and may be a trait of the psychotic condition. Stratification of patients based on oxidative-related parameters, according to precision medicine, is a promising approach to increasing SZ therapy's effectiveness/to facilitating a new approach based on precision medicine and implementing biological/biochemical-tailored treatment to restoring redox balance (11, 52).

## Limitations

The study has limitations that should be acknowledged. The assessment included only peripheral blood parameters. Further studies should concentrate on analyzing them together with neuroimaging assessment to detect the relationship between central and peripheral oxidative status. The anthropometric values (BMI) were different between examined groups, which can strongly affect oxidative parameters (53). Nevertheless, in our analysis, BMI not affect most of the assessed biomarkers. The individuals from patients' groups had not taken the same type of antipsychotic drug. To control for patient heterogeneity, we performed analysis according to the illness stage and adjusted obtained results of potential cofounders. However, the treatment and drug interaction with intra-individual variability could change pro/antioxidative response in many different manners.

Another limitation is the lack of correlation between OS and critical biological factors specific for the SZ process, progression, or stage (54–56). Determining the relationship between them and the OS biomarkers is an urgent need. In clinical practice, based on psychopathological symptoms, severity assessment on the clinical interview is treated as a gold standard in SZ diagnosis. Some authors, based on the preclinical studies, suggest some of the SZ biomarkers. Up to date, the information and results of these studies are limited. This point needs further investigation.

It is well-known that antioxidants are very labile molecules. Only in the presence of damage initiated, for example, at the initial or terminal stage, can their activity be linked to specific directions of OS shifts. However, we did not assess these stages. In further exploration, more attention should be paid to another critical indicator of OS that we omitted (i.e., lipid peroxidation or thiobarbituric acid active products). Measurement of them would provide important information about the levels of the processes being studied.

## Conclusions

The study results confirm differences in serum levels of OS biomarkers between patients suffering from SZ and healthy individuals. In patient groups, the levels of oxidation products and antioxidant depend on the stage of illness (first-episode patients vs. chronic SZ). The pro/antioxidant status could be considered a predictor of SZ (CAT, GPX, TAC, FRAP, AGEs, and AOPP) or/and factor affecting patients' symptom severity (CAT, FRAP, KYN, NFK, DITYR). Monitoring of peripheral blood level oxidative biomarkers is a promising method in the SZ approach. However, critical analysis including more confounding factors is required to confirm obtained results.

## Data Availability Statement

The original contributions presented in the study are included in the article/Supplementary Material, further inquiries can be directed to the corresponding author/s.

## Ethics Statement

The studies involving human participants were reviewed and approved by Ethics Committee of the Medical University of Lublin, Poland. The patients/participants provided their written informed consent to participate in this study.

## Author Contributions

DJ, MD, and HK-J: conceptualization. JR: data curation and formal analysis. DJ, MD, NW, AZ, and MM: investigation and methodology. HK-J: project administration and supervision. DJ, JR, and HK-J: writing—original draft. DJ, JR, and HK-J: writing—review and editing. All authors contributed to the article and approved the submitted version.

## Conflict of Interest

The authors declare that the research was conducted in the absence of any commercial or financial relationships that could be construed as a potential conflict of interest.

## Publisher's Note

All claims expressed in this article are solely those of the authors and do not necessarily represent those of their affiliated organizations, or those of the publisher, the editors and the reviewers. Any product that may be evaluated in this article, or claim that may be made by its manufacturer, is not guaranteed or endorsed by the publisher.
