# Supplementary material for: Oxidative Stress Biomarkers as a Predictor of Stage Illness and Clinical Course of Schizophrenia
Source: Front Psychiatry. 2021 Nov 15;12:728986. doi: 10.3389/fpsyt.2021.728986 (PMC8636114; doi:10.3389/fpsyt.2021.728986)
Supplement: Supplementary file 1 [file Table_1.DOCX]

Supplementary Material

# Supplementary Data

**
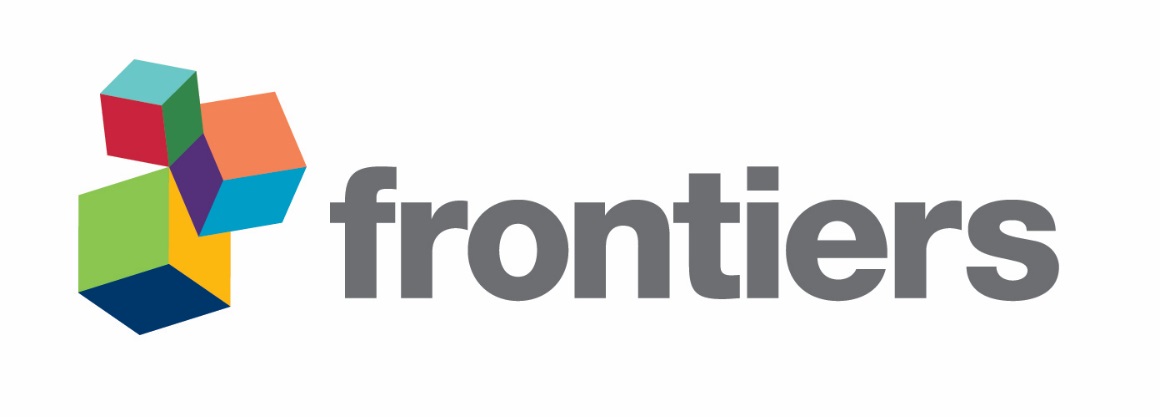
**

**Supplementary Table 1.**Correlation between oxidative stress and examined factors in study population

| **Group  Variables** | **SZ** | **FS** | **CS** | **HC** |
| --- | --- | --- | --- | --- |
| **CAT** | NoC (-0.23) DoI (-0.29) NoE (-0.31) PANSS_n (-0.34) | NoC (-0.32) DoI (-0.38) NoE (-0.29) PANSS_n (-0.40) | NA | NA |
| **SOD** | NoC (-0.28) BMI (-0.29) Age (-0.49) DoI (-0.47) NoE (-0.39) PANSS_p (0.35) PANSS_g (0.33) PANSS (0.32) | Age (-0.32) NoC (-0.43) | NA | BMI (-0.55) |
| **GR** | DoI (0.35) NoE (0.31) | NA | NA | BMI (0.42) |
| **TAC** | Age (0.42) DoI (0.47) NoE (0.37) PANSS_p (-0.38) PANSS_g (-0.35) PANSS (-0.32) | NoC (0.36) | DoI (0.33) | NA |
| **OSI** | NoC (-0.30) | (-0.48) | BMI (0.41) PANSS_n (-0.32) | NA |
| **FRAP** | NA | PANSS_p (0.34) | NA | NA |
| **AGEs** | NA | PANSS (0.33) | NA | NA |
| **AOPP** | NA | NA | Age (-0.39)  DoI (-0.35) | NA |
| **DITYR** | Age (-0.35) PANSS_p (0.29) PANSS_g (0.31) PANSS (0.31) | NA | BMI (-0.33) | NA |
| **KYN** | BMI (-0.33) Age (-0.50) DoI (-0.48) NoE (-0.38) PANSS_p (0.43) PANSS_g (0.39) PANSS (0.38) | NA | NA | NA |
| **NFK** | BMI (-0.26) Age (-0.31) DoI (-0.30) NoE (-0.26) PANSS_p (0.25) PANSS (0.25) | PANSS_n (0.39) PANSS (0.35) | NA | NA |
| **NO** | DoI (0.26) | NA | NA | NA |

SZ – schizophrenia; FS – first episode; CS – chronic schizophrenia; HC – healthy control. CAT – catalase; GPx - glutathione peroxidase, SOD-1 - superoxide dismutase-1; GR - glutathione reductase; GSH - reduced glutathione ; TAC - total antioxidant capacity; TOS – total oxidant status; OSI - oxidative stress index; FRAP - ferric reducing ability of plasma; AGEs - advanced glycation end products; AOPP - advanced oxidation protein products; DITYR – ditirosine, KYN – kynurenine; NFK - N-formylkynurenine; TRY - tryptophan; NO - nitric oxide; DoI – duration of ilness; NoE – number of episodes, PANSS – Positive and Negative Symptoms Scale: p – positive subscale/n – negative subscale/g – general subscale; BMI – body mass index; NoC – number of cigarettes per day; NA – not applicable. Only statistical significant results (p<0.05) of Spearman’s rank correlation coefficients are presented (R)
